# The use of personalized behavioral feedback for online gamblers: an empirical study

**DOI:** 10.3389/fpsyg.2015.01406

**Published:** 2015-09-23

**Authors:** Michael M. Auer, Mark D. Griffiths

**Affiliations:** ^1^neccton ltd.Lienz, Austria; ^2^Department of Psychology, Nottingham Trent UniversityNottingham, UK

**Keywords:** responsible gambling, player tracking, problem gambling, harm minimization, player protection

## Abstract

Over the last few years, online gambling has become a more common leisure time activity. However, for a small minority, the activity can become problematic. Consequently, the gambling industry has started to acknowledge their role in player protection and harm minimization and some gambling companies have introduced responsible gambling tools as a way of helping players stay in control. The present study evaluated the effectiveness of *mentor* (a responsible gambling tool that provides personalized feedback to players) among 1,015 online gamblers at a European online gambling site, and compared their behavior with matched controls (*n* = 15,216) on the basis of age, gender, playing duration, and theoretical loss (i.e., the amount of money wagered multiplied by the payout percentage of a specific game played). The results showed that online gamblers receiving personalized feedback spent significantly less time and money gambling compared to controls that did not receive personalized feedback. The results suggest that responsible gambling tools providing personalized feedback may help the clientele of gambling companies gamble more responsibly, and may be of help those who gamble excessively to stay within their personal time and money spending limits.

## Introduction

In recent years, online gambling has become a more common leisure time activity. Data from 2010 British Gambling Prevalence Survey reports that 14% of the population gambled on the internet in the past year (Wardle et al., 2011a). According to Griffiths (2003), there are a number of situational and structural characteristics that make online gambling potentially risky for susceptible and vulnerable individuals such as problem gamblers. Such factors include accessibility, affordability, anonymity, and specific structural features of online games such as high event frequency. Some forms of online gambling may be more problematic than others. For instance, playing online poker (rather than games of pure chance such as an online bi-weekly lottery) has been linked to problematic gambling in some players (e.g., Griffiths et al., 2010). Problem gambling typically refers to gambling that leads to social, psychological, and/or financial difficulties that compromise areas of gamblers’ lives such as their job, education, personal relationships, health, etc. (Griffiths, 2004).

A number of empirical studies have observed that there are typically more problematic gamblers among those that gamble on the internet compared to those that only gamble in land-based venues. (e.g., Wood et al., 2007; Griffiths and Barnes, 2008; Griffiths et al., 2009; Wood and Williams, 2011; Gainsbury et al., 2014). However, it should also be noted that problem gambling severity is associated with overall engagement and that when the volume of gambling is controlled for, online gambling is not predictive of problems (Philander and MacKay, 2014). Furthermore, most online gamblers are also oﬄine gamblers and gamble on many different activities and across different gambling platforms (Wardle et al., 2011a).

### Messaging and Feedback Tools in Responsible Gambling

Given the increasing number of people gambling online and issues surrounding problem gambling, many of the more socially responsible gambling companies around the world have started to use responsible gambling tools to help their clientele gamble more responsibly. Consequently, gamblers can now access and/or are given general advice on healthy and responsible gambling, as well as information about common misbeliefs and erroneous perceptions concerning gambling^1^^,^^2^^,^^3^ (Gaboury and Ladouceur, 1989; Griffiths, 1994; McCuster and Gettings, 1997; Parke et al., 2007; Wohl et al., 2010). However, findings on the effectiveness of providing gamblers with information in correcting or changing erroneous beliefs have been mixed. Some outcomes support the display of information (Dixon, 2000; Ladouceur and Sevigny, 2003) while other studies have reported non-significant results (Hing, 2003; Focal Research, 2004; Williams and Connolly, 2006).

A small body of empirical research has shown that educational programs about erroneous beliefs can successfully help change the targeted cognitions (e.g., Wulfert et al., 2006; Wohl et al., 2010). For instance, Wohl et al. (2010) developed an animation-based educational video regarding the function of slot machines. Results demonstrated that participants increased behavioral intentions to use strategies to stay within limits and reduce frequency of exceeding limits. The authors also showed that animated educational information on slot machines can be an effective way to increase user adherence to preset (i.e., predetermined) monetary spending limits.

Studies have also shown that the way information is presented can significantly influence behavior and thinking. Several studies have investigated the effects of interactive versus static pop-up messages during gambling sessions. Static messages do not appear to be effective, whereas interactive pop-up messages and animated information have been shown to change both irrational belief patterns and behavior (e.g., Schellink and Schrans, 2002; Ladouceur and Sevigny, 2003; Cloutier et al., 2006; Monaghan and Blaszczynski, 2007, 2010a; Monaghan et al., 2009). Monaghan and Blaszczynski (2010a) found that pop-up messages on electronic gambling machines (EGMs) containing self-appraisal messages had significant effects on self-reported thoughts and behavior during the experimental sessions.

Monaghan and Blaszczynski (2010b) also asserted that informational warning signs should promote the application of self-appraisal and self-regulation skills rather than the simple provision of information. Stewart and Wohl (2013) reported that participants who received a monetary limit pop-up reminder were significantly more likely to adhere to monetary limits than participants who did not. Wohl et al. (2013) examined two responsible gambling tools that targeted adherence to monetary limits among 72 EGM gamblers. These tools comprised an animation-based educational video (used previously by Wohl et al., 2010) and a pop-up message. Consistent with previous findings, both types of responsible gambling tools achieved the effects they were intended to do. More specifically, the findings showed that a pop-up limit reminder helped gamblers stay within their pre-determined monetary limits. Another study by Munoz et al. (2013) demonstrated that graphic warning signs were more effective than text-only warnings in changing attitudes and complying with the warning signs.

Auer et al. (2014) investigated the effect of a pop-up message that appeared after 1,000 consecutive online slot machine games had been played during a single gambling session. The study analyzed 400,000 gambling sessions (200,000 sessions before the pop-up had been introduced and 200,000 after the pop-up had been introduced). The study found that the pop-up message had a limited effect on a small percentage of players. Although the study reported nine times as many gamblers stopped after 1000 consecutive plays compared to those gamblers before the introduction of the pop-up message, the number of gamblers that actually stopped after viewing the pop-up message was less than 1%.

In a follow-up study, Auer and Griffiths (2015) investigated the effects of normative and self-appraisal feedback in a slot machine pop-up message compared to a simple (non-enhanced) pop-up message. The study compared two representative random samples of 800,000 gambling sessions (i.e., 1.6 million sessions in total) across two conditions (i.e., simple pop-up message versus an enhanced pop-up message). The results indicated that the additional normative and self-appraisal content doubled the number of gamblers who stopped playing after they received the enhanced pop-up message (1.39%) compared to the simple pop-up message (0.67%). Like the previous study, the findings suggested that pop-up messages influence only a small number of gamblers to cease long playing sessions but that enhanced messages are slightly more effective in helping gamblers to stop playing within-session. These two studies (i.e., Auer et al., 2014; Auer and Griffiths, 2015) are the only studies to examine the impact of messaging on actual gamblers in a real world online gambling environment.

### Personalized Feedback

Personalized behavioral feedback has been studied in other areas outside of the gambling studies field that involve potentially addictive behavior (e.g., tobacco and smoking research). For instance, Stotts et al. (2009) found that motivational interviewing along with ultrasound feedback was effective for pregnant light smokers. Obermayer et al. (2004) targeted college students using integrated internet and cell phone technologies to deliver a smoking-cessation intervention. Their findings provided support for using wireless text messages to deliver potentially effective smoking-cessation behavioral interventions to this particular group of people.

Outside of the addiction studies field, Cho et al. (2009) studied the effects of personalized behavioral feedback in the management of Type 2 diabetes. Here, Type 2 diabetes patients used mobile phones that automatically measured glucose levels and transferred this information to the internet. The authors found that web-based charts displaying individual data and personalized feedback in the form of text messages were effective means for decreasing the glucose level in diabetes sufferers. A similar study by Farmer et al. (2005) described a real-time system for Type 1 diabetes patients. Their telemedicine system collected real-time information about glucose level as well as information regarding insulin dose, eating patterns, and physical exercise. The system gave verbal and illustrated feedback so that patients could better keep track and control their glucose level. The feedback led to regular maintenance of blood glucose level and an increased number of patients met their predetermined targets. Another area where behavioral feedback has been investigated is in the area of sports and fitness. Buttussi et al. (2006) investigated the use of mobile phone guides in fitness activities using a Mobile Personal Trainer (MOPET) application. The mobile app gave verbal navigation assistance and also used a 3D-animated motivator. Evaluation of the results supported the use of mobile apps and embodied virtual trainers in outdoor fitness applications.

The approaches outlined above aim to change a person’s behavior via behavioral feedback. Such approaches are based on the ‘stages of change’ model (Prochaska and DiClemente, 1983; Prochaska and Prochaska, 1991) and motivational interviewing (Miller and Rollnick, 1991). Therefore, in order to change people’s gambling behavior using behavioral tracking data, the present authors believe that player feedback should also be presented in a motivational way and take into account the stages of change model. In practical terms, this means presenting messages in a non-judgmental way alongside normative data so that gamblers can evaluate their actions compared to other like-minded individuals. The importance of non-judgmental and transparent feedback is also underlined by studies from other areas. A study by Lapham et al. (2012) on alcohol drinking advocated transparent feedback, as their participants were almost unanimous in their wish to know how they were assigned to their particular risk category for alcohol drinking. They suggested that tailored personal feedback could include a summary of a participant’s drinking and whether, and the extent to which, weekly or daily limits were exceeded.

### Human–Computer Interaction and Persuasive System Design

The main goal of pre-commitment tools is to change human behavior and yet their designs have only recently been linked to the principles of human–computer interaction (HCI) and persuasive system design (PSD). Wohl et al. (2014) found a HCI and PSD inspired monetary limit pop-up tool to be significantly more effective compared to a tool that did not incorporate these principles. HCI is a field of research that investigates the interaction of people with interactive technology and tries to increase usability and uptake. Persuasive Technology has been defined as interactive computing systems designed that attempt to change people’s attitudes and behaviors (Fogg, 2003). Apart from user-feedback, HCI principles relevant for the design of pre-commitment measures are an esthetic visual design, the incorporation of system-status updates, a sense of control over functionality, and the use of simple language (Hewett et al., 1992; Shneiderman et al., 2009; Preece et al., 2011). Apart from showing that messaging can effectively change thoughts about gambling and the gambling behavior itself, research has also suggested that the content of messages is important (Monaghan and Blaszczynski, 2010a,b). Along with Wohl et al. (2014), the present authors argue that the design of a responsible gambling feedback system is crucial, and that HCI and PSD principles can facilitate and improve the effect.

Fogg (2003) outlined seven types of persuasive tools in designing systems that intend to motivate attitude or behavior change. These are: (i) *reduction* which states that tasks should be as simple as possible, (ii) *tunneling* in which users should be led through a series of steps to achieve their goals, (iii) *tailoring* in which users are provided with specific design and information, (iv) *suggestions* which describe interventions at the right moment to suggest action, (v) *self-monitoring* which empowers users to monitor their own progress toward achieving a desired attitude or behavior, (vi) *surveillance* which allows an external party to monitor user behavior with the intent to motivate change, and (vii) *conditioning* which employs principles of operant conditioning to bring about change. The principles of PSD have been successfully applied to various domains including obesity (Toscos et al., 2006; Tsai et al., 2007), Borderline Personality Disorder (Rizvi et al., 2011), smoking cessation (Lehto and Oinas-Kukkonen, 2009), and alcoholism (Lehto and Oinas-Kukkonen, 2009; Cohn et al., 2011).

The present study examined personalized feedback and information given to players during real world gambling sessions. More specifically, its aim was to investigate the effects of personalized information about past gambling behavior on future gambling. It was hypothesized that gamblers receiving tailored feedback about their online gambling behavior would be more likely to change (i.e., reduce) their behavior (as measured by the amount of time and money spent) compared to those who did not receive tailored feedback. The null hypothesis was that gamblers receiving tailored feedback about their online gambling behavior would show no reduction in their gambling behavior (as measured by the amount of time and money spent) compared to the control group.

## Materials and Methods

### Participants

This is a secondary data analysis study using quantitative data provided to the authors by the gambling operator. The researchers were given access to the behavioral tracking data of 1,358 gamblers at a European online gambling website that had voluntarily signed up to a behavioral feedback system (*mentor*) that is offered to all customers on the website. The behavioral feedback system is an additional service provided by the gambling operator. The players were notified about the system via email and they also had information available online while they are playing. The participants were not selected randomly as they could decide for themselves whether to opt into using the service that was advertised on the gambling website as a responsible gambling tool that helps players gamble more responsibly.

### Overview of the Behavioral Feedback System

**FIGURE 1:**
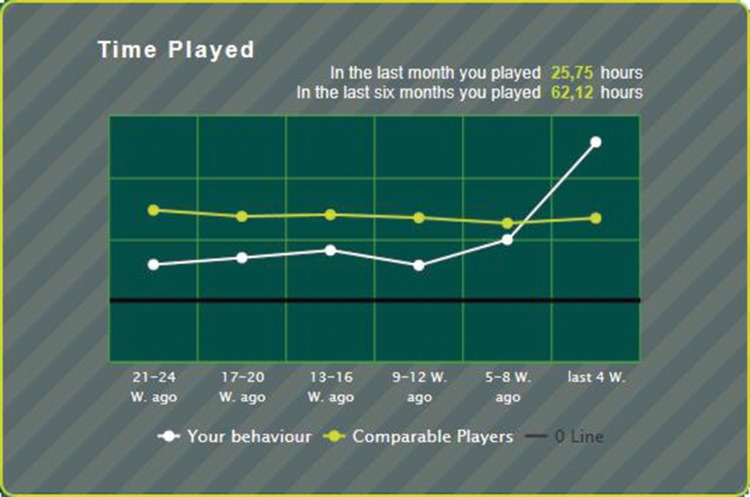
**Illustration of ‘time played’ information in the feedback system as seen by a hypothetical player**.

This section provides a brief description of the behavioral feedback system implemented by the gambling operator. The system is an opt-in system (i.e., gamblers can voluntarily choose to use it and the system is not mandatory). Once gamblers have enrolled to use the system, they can retrieve detailed visual and numerical feedback about their gambling behavior via a button on the website. Player feedback is displayed in a number of ways (numerical, graphical, and textual) and provides information about wins and losses, playing duration (PD), number of playing days, and games played. The system can also display personal gambling behavior over time. For instance, **Figure 1** shows the playing time information for a hypothetical player in the form of a graph over time. At the top of the screen, players receive information about playing time over the previous 4-week and 24-week period. The white line in **Figure 1** indicates that the player shows an upward trend and is steadily increasing the amount of time spent gambling. During the previous 4-week period, the player spent 25.75 h gambling online. The upper line in **Figure 1** is the average playing time for all other comparable (either lottery-type or casino-type) online players and provides the gambler both normative and comparative feedback. Such feedback has been emphasized as an important aspect in facilitating behavioral change (Miller and Rollnick, 1991). Players are either assigned to ‘lottery’ type players or ‘casino’ type players based on their playing patterns. The categorization is derived from the theoretical loss (TL) that is produced in casino and lottery games respectively.

Of the daily active players, 10% (*n* = 1,358) opted into the system. Players could opt-in via a clearly visible button on the post-login website page which appeared immediately after they logged into their account. The personalized information appeared in a new pop-up window. This typically led to a break in play, as gamblers who viewed the information are unlikely to play and view information simultaneously. Due to reasons of data protection, the players’ interaction with the system is anonymous and not tracked. For this reason it is not known how often players retrieve the information or how much time they spent viewing the information. The system tracks those players who sign up and therefore the opt-in date is known and can also be used for analytical purposes.

**FIGURE 2:**
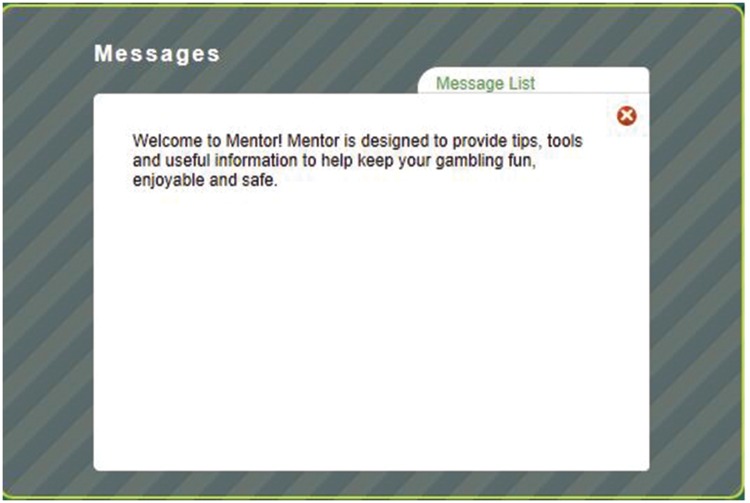
**Information message sent to players about the feedback system**.

Game categories were developed similar to other research in the gambling studies field (Auer et al., 2012; Gainsbury et al., 2012). The eight game types available on the gambling operator’s website are Lottery Draw, Lottery Instant, Poker, Bingo, Casino Slots, Casino Videopoker, Casino Table, and Sports Wagering. Additionally, players receive a message that welcomes players to the system (see **Figure 2**). All the visual, numerical, and textual information can be accessed by the gambler via a user-friendly on-screen dashboard. Responsiveness means that interactive content automatically adapts to technical environments. The player front end thus looks similar on different devices such as desktops, laptops, mobile phones, or tablets and also across different browsers and operating systems such as *Windows, Android*, or *iOS*. In line with HCI principles, professionals both from a content and visual point of view designed the system. PSD was taken into account by the salient self-monitoring features of the system, the personalized content, and normative information.

The hypothesis investigated whether players’ gambling behavior (i.e., time and money spent gambling) changes after they have registered for the system and see the personalized feedback for the first time compared to the gambling behavior of a matched pairs control group. Both PD and TL 14 days before and 14 days after registration were measured. TL refers to the amount of money wagered multiplied by the payout percentage of a specific game played. In order to be able to investigate the effect of the personalized feedback an appropriate time period of playing had to be observed. If there was an influence on gambling behavior it would most certainly materialize quickly after the information had been viewed. If a short-term change in gambling behavior is visible, long-term changes can be hypothesized and investigated in future studies. A long-term change was unlikely and would only have been observable in a true experimental setting.

The next issue was determining the length of time needed to detect behavior change. The distribution of gambling behavior on the gambling website ranges from daily play to weekly play to less than weekly play. Additionally, players were not randomly assigned to the study and therefore several other factors could not be controlled for. The online gambling site imposes a weekly deposit limit upon all players that cannot be exceeded. There are also numerous marketing campaigns that target players at any given time. For that reason, a longer observable time period would probably not have yielded any significant changes as the gambling behavior may have been influenced by many other factors. However, if the time period is chosen too short, changes might only be purely random and players who play rarely might not even have had a chance to play. For that reason the authors chose to compare 14 days of playing behavior prior to opt-in to 14 days after opt-in. Two metrics – ‘TL’ and ‘PD’ were measured.

Theoretical loss is a concept that was developed by Auer et al. (2012), and has been empirically shown as a robust and stable measure of monetary gambling intensity. For instance, an empirical study by Auer and Griffiths (2013b) showed that the TL is a much more accurate indicator of monetary gambling intensity than proxy measures such as bet size and the number of bets made. This is especially important when gambling behavior is investigated across different game types such as the present study.

### Rationale for Matched Pairs Design

The aim of the present study was to determine whether the presentation of personalized feedback to gamblers has an effect on their subsequent playing behavior compared to those gamblers that do not receive personalized feedback. Due to the fact that the players voluntarily chose to sign up for the service it is not appropriate to simply compare the behavior before and after the registration, as the sample is not a random representation of the population.

After the data were provided, the present authors gave very carefully thought about all of the ways in which the data could be analyzed. Following an initial inspection of the data, it became clear that comparing the overall amount of time and money spent by gamblers before and after using the personalized feedback system (i.e., within-group analysis) would not be meaningful because there was very large variation in what individual gamblers spent financially and how long they played in terms of time. For instance, some gamblers spent 100s of Euros on every gambling session while others spent just a few Euros per session. The resulting mean average differences in terms of time and money spent as a whole group before and after using the personalized feedback tool were therefore likely to be spurious because of the large individual differences in gambling behavior. Futhermore, there was no way of assessing whether the difference in the amount of time and money spent within group was significant as there was no reliable comparison point. Therefore, a control group was needed.

One way to determine a valid control group is via a matched pairs design in which similar players out of the population are assigned to each of the 1,358 target group members. The control group population only comprised online gamblers that had not used the system but who played during the period in which those in the target group signed up for the system. Matched pairs for the target group members were chosen using the following criteria:

– **Age**: Control group members had to be in the same age group as the target group member. Age groups were derived from Wardle et al. (2011a).
– **Gender**: Control group members had to be the same gender as the target group member.
– **Playing duration 14 days before registration**: Control group members had to have gambled for the same amount of time as the target group. Players were matched if their PD in the 14 days before the registration date was within 10% of the target individual. For instance, if a target group member played for 10 h during the 14 days, the control group member’s PD needed to be within 9–11 h in order to be considered for matching.
– **Theoretical loss 14 days before registration**: Control group members had to have the same TL as the target group. Players were matched if their TL in the 14 days before the registration date was within 10% of the target individual. Control group members were matched if their TL in the 14 days prior to registration was within 10% of the target individual. For instance, if a target group member’s TL was €100, the control group member’s TL needed to be within €90–€110 in order to be considered for matching.

Demographic variables have reported to correlate with gambling behavior. Potenza et al. (2001) reported gender-related differences in underlying motivations to gamble and in problems generated by excessive gambling. They concluded that different strategies may be necessary to maximize treatment efficacy for men and for women with gambling problems. Frequency of play as measured via ‘PD’ and ‘TL’ are important moderators of gambling behavior. Afifi et al. (2014) re-analyzed the data from the nationally representative Canadian Community Health Survey (CCHS) and showed that after adjusting for gambling involvement, gender and age no longer moderated the correlation between frequency of play and game type. They suggested a shift toward a more complex model that also included the level of gambling involvement. The type of game played was not used to match players. However, similar values in PD as well as TL will necessarily lead to similar game-type preferences. It is unlikely that a player who spends a lot of time on slot machines would be matched with a player who preferred lottery games, because lottery players do not require much interaction with the website to gamble.

All of the four main criteria (age, gender, PD, TL) were weighted equally. For that reason, each target group member was matched with at least one control group member (as described above). On the majority of paired matches, target population individuals were paired with more than one control player from the total population that amounted to ∼53,000 players. In order to determine the effect for each target group member, PD and TL in the 14 days after the registration were divided by the PD and TL 14 days before the registration. This indicator will subsequently be called the ‘ratio.’

For each gambler that used *mentor* and for each gambler who did not, the ratio of playing intensity was computed as well as PD before and after they signed up to the system. The smaller the ratio, the lower the subsequent gambling intensity (in terms of PD and TL), and therefore higher the effect of the personalized feedback. Each target group member’s computed ratio was compared to the mean average ratio of the matched pairs for that target group member, both for PD and TL. If a target group member’s ratio was smaller than the respective control group’s average ratio it was concluded that the target group member’s behavior decreased more as a consequence of the personalized feedback compared to the control group members who did not receive this information. So for each target/control pair, a binary variable was computed. The actual difference was not analyzed as the different target/control pairs showed large individual variation. The way the study was designed was to make sure the gambling behavior between the two groups were comparable (that is why the matched pairs design was chosen). Ethical approval for the study was given by the research team’s University Ethics Committee.

## Results

### Gamblers Using the Personalized Feedback System

Of the 1,358 gamblers that had registered to use the *mentor* system, the vast majority (*n* = 1,119) had played on the website in the 14 days prior to their registration on the system. The 239 gamblers that did not gamble 2 weeks before registering were excluded from the analysis. This was because it would be impossible to determine if the behavioral feedback had an effect on subsequent behavior because the starting point would have been no gambling activity (meaning they would have automatically showed an increase in gambling intensity).

### Gender Distribution of Samples

The gender distribution in the target group was compared with the expected gender distribution that was computed from all active players during the research period. Via this comparison, the representativeness of the target group to the whole population on the gambling website with respect to gender can be determined. The distribution of 80% males and 20% females in the target group did not deviate significantly from the expected distribution of 78% males and 22% females. The chi-square test was not significant (χ^2^[1] = 1.22, *p* = 0.27). Therefore, in terms of gender, the target group was representative of the population of players on that gambling website.

### Age Distribution of Samples

The age distribution in the target group was compared with the expected age distribution that was computed from all active players during the research period. The chi-square test was significant (χ^2^[6] = 46.24, *p* < 0.0001) which means that in terms of age, the target group was not representative of the population of players. The biggest difference occurred in the group aged 30–44 years. In the target group, 18% were between 30 and 44 years, and in the population group, 25% were between 30 and 44 years. No other differences were observed.

### Gambling Intensity of Samples

In order to determine the target groups’ representativeness regarding gambling intensity, the TL percentile values at 10, 25, 50, 75, and 90% in the population of active players were determined. The number of target group members between those percentiles was then computed. **Table 1** contains the distribution of target group members with respect to TL. The column ‘expected %’ describes the expected percentages according to the population distribution. It can clearly be seen that the sample is underrepresented in the lower range and overrepresented in the higher range. For instance, 17% of the target group members are in the top 10% whereas only 10% would have been expected if the target group was equally distributed across the population. On the other hand, only 18% of the target group members are between the first quartile (Q1) and the median, whereas 25% would be expected if the target group was equally distributed across the population. Consequently, the chi-square analysis was significant (χ^2^[5] = 134.22, *p* < 0.0001).

**Table 1:** Theoretical loss (TL) distribution of the online gambler target group population (*n* = 1,119).

	*N*	Actual %	Expected %
10th percentile	87	8%	10%
25th percentile	106	9%	15%
Median	200	18%	25%
75th percentile	307	27%	25%
90th percentile	224	20%	15%
100th percentile	195	17%	10%
	1,119		

### Group Differences

The differences in age and TL between the target group and the population of all active players are important indicators for the necessity of a matched pairs design described above. Therefore, each target group member was matched with several control group members that did not opt to use the behavioral feedback system. As all the indicators for matching were weighted equally, all control group members that met the required criteria were selected for a given target group member. At least one valid control group member was found for 1,015 of the players that had registered for the system. Therefore, 104 target group members where discarded from the analysis because of a lack of comparability. **Table 2** displays the distribution of the number of matched control group members across the remaining 1,015 individuals in the target population. On average, each target group member was matched with 18 control group members. The same control group members were sometimes matched with several different target group members and the total number of unique online gamblers in the control group was 15,216. This number is reported in **Table 2** as “N unique control.” The maximum number of control group members matched with one target group member was 260.

**Table 2:** Distribution of the number of matched controls across the target group.

*N* (target)	1,015
Min	1
Max	260
Average	17.99
*SD*	25.52
N unique control	15,216
10th percentile	2
25th percentile	4
Median	9
75th percentile	20
90th percentile	48

### Effect of Personalized Feedback

The effect that the personalized behavioral feedback had on subsequent TL and PD of those that signed up to the system was then statistically analyzed and compared with that of the control group. It was assumed that any difference between the gambling behaviors in the two groups could be due to chance and would be similar to the tossing of a coin. For that reason, it was assumed under the null hypothesis that in 50% of the cases the target group’s gambling behavior (as measured by time and money spent) would be higher than the control group’s gambling behavior and in 50% of the cases the control group’s gambling behavior (as measured by time and money spent) would be higher than the target group’s gambling behavior. Therefore, any deviation from that distribution is due to the effect of the tailored feedback. In the present study, the difference between the actual observed percentage to the expected percentage (50%) of gambling behavior was statistically tested.

Of the 1,015 target group members, 625 (62%) showed a smaller TL ratio and 610 (60%) showed a smaller PD ratio (compared to the average TL ratio of the matched control group members). Among these target group members, overall gambling behavior (as measured by TL and PD) decreased more after registration than among the matched control group members. A standard normal distribution test was used to compare the actual percentage of target group members who showed a smaller TL than the respective control group members with the expected percentage of target group members who showed a smaller TL than the respective control group members. The results showed significant differences for both TL (*Z* = 7.38; *p* < 0.0001) and PD (*Z* = 6.43, *p* < 0.0001). Therefore, behavioral feedback had the desired impact on subsequent playing behavior with respect to monetary spending and play duration.

### Personalized Feedback and Gambling Intensity

Analysis was also carried out to see if gambling intensity was associated with the effect of personalized feedback. To do this, the 1,015 target group members were divided into ten equally sized groups according to the TL in the 14 days prior to registration. **Table 3** shows the percentage of target group members in each group for which the TL or PD ratio was smaller than the average of the matched control group members. The 10% least gambling intense players (as measured by TL) had the lowest effect on the time and money they spent gambling, whereas those in the fourth group had the highest effect (see **Table 3**). However, no clear pattern emerged.

**Table 3:** Effect of the behavioral feedback on TL and playing duration (PD) across monetary gambling intensity groups.

TL group	Effect TL %	Effect PD %
1	55	51
2	56	59
3	56	53
4	74	74
5	62	61
6	60	53
7	65	70
8	66	59
9	62	58
10	59	62

One would naturally expect that an intervention such as the responsible gambling tool in this study would influence the time and money spent in a similar way. More specifically, players that decreased the amount of money they spent would also be more likely to decrease the amount of time they spent gambling (and vice versa) as a consequence of using the system. If there was no association between the changes in time and money spent then it would likely indicate potentially spurious results that might have occurred purely by chance. Consequently, to further evaluate the internal validity of the results, the association between the effects on time and money spent across the target group members was statistically examined. This was done via cross-tabulating the effect on TL and PD (see **Table 4**). In order to determine if there is a positive association between TL and PD, the frequencies expected under the null hypothesis were computed. The expected frequencies under the null hypothesis are highlighted in **Table 4**.

**Table 4:** Observed and expected cross-table between effect on TL and PD.

Effect TL	No	Yes	
**Observed Effect PD**		
No	289	101	390
Yes	116	509	625
	405	610	1015

**Expected Effect PD**		
No	156	234	390
Yes	249	376	625
	405	610	1015

Comparing the observed and expected frequencies in **Table 4** it can be seen that the values in the lower half of the table are bigger in in the main diagonal and smaller in the secondary diagonal. This means that the number of players who showed an effect in relation to time and money spent was bigger than expected (i.e., 289 vs. 156) and the number of players that did not show an effect in relation to time and money spent was also bigger than expected (509 vs. 376). The number of players that only showed an effect in one of the behaviors was smaller than expected. This means that the registration influenced time and money spent in a similar way and underlines the internal validity of the study. Given the fact that the main diagonal’s numbers are higher than expected under the null hypothesis and the secondary diagonal’s numbers are lower than expected, it is not surprising that the distribution in the lower half **Table 4** is significantly different from a purely random distribution (χ^2^[1] = 309, *p* < 0.0001).

## Discussion

The present study aimed to evaluate the effectiveness of a personalized behavioral tool (i.e., *mentor*) on subsequent gambling behavior in a real world population of online gamblers by comparing it with a group of matched controls that did not receive personalized feedback. Results indicated that the personalized feedback system achieved the anticipated effect and that the time and money spent gambling was significantly reduced compared to that of the control group. The results suggest that responsible gambling tools such as *mentor* may help the clientele of gambling companies gamble more responsibly, and may be of help those who gamble excessively.

Although the present study did not study disordered (i.e., problem) gambling, responsible gambling tools may also be of help to this group of gamblers. Disordered gambling may be influenced by the failure to set and adhere to pre-set monetary limits (Lesieur, 1979). Tools such as the system evaluated here, may help facilitate the setting of and adhering to such limits as some of the personalized information provided links to pre-commitment limit setting tools on the gambling operator’s website. Pre-commitment measures have been shown to effectively limit players’ time and money spent gambling (Auer and Griffiths, 2013a; Stewart and Wohl, 2013; Wohl et al., 2013). The results in the present study appear to concur with the literature on dynamic pop-up messages that show they effectively change players’ gambling-related beliefs and subsequent behavior (e.g., Schellink and Schrans, 2002; Ladouceur and Sevigny, 2003; Cloutier et al., 2006; Monaghan and Blaszczynski, 2007, 2010a; Monaghan et al., 2009). The findings also support the assertions of Monaghan and Blaszczynski (2010b) who claimed that warning signs should promote the application of self-appraisal and self-regulation skills.

To the authors’ knowledge, this is the first real world online gambling study that has investigated the effects of behavioral feedback on actual gambling behavior within a real online gambling website. The study takes into account many of the findings from previous research, such as presenting information in a non-confrontational way (e.g., Miller and Rollnick, 1991) and displaying them in an appealing and HCI-inspired interactive environment (Wohl et al., 2010, 2014). In the present study, players received personalized information along with normative comparisons that reflected their actual personal gambling behavior.

The way that the information was presented was in line with previous laboratory research and followed concepts of HCI and PSD principles (Fogg, 2003; Wohl et al., 2010, 2014). One of the goals of the study was to investigate whether personalized numerical and visual (as well as normative) feedback could change behavior (i.e., reduce the amount of time and money spent gambling) in a real world gambling environment. Results showed that compared to the control group, players significantly decreased the amount of time and money spent after they were exposed to the personalized information about their individual behavior for the first time. These results appear to show that personalized, behavioral feedback has significant and relatively immediate effects on subsequent gambling behavior compared to controls. This is not surprising given the evidence in the gambling studies field (e.g., Auer and Griffiths, 2013a; Kim et al., 2014) as well as other areas of non-gambling research (Farmer et al., 2005; Buttussi et al., 2006; Cho et al., 2009; Colkesen et al., 2013). The main results were also validated by additional analysis showing that the individual players reacted similarly with respect to time and money spent when provided with personalized feedback.

Despite the many strengths of this study, there are a number of key limitations. All of the participants in the target population had voluntarily registered to use the system and were therefore not selected randomly from the population of players. In an attempt to overcome this, a matched pairs design was chosen in which each and every target group member was matched with a number of most similar control group members who were not given personalized feedback. This matched pairs design is the next best approach in overcoming the problems associated with investigating non-randomly selected target group members. However, it is worth noting that in reality, most responsible gambling tools and systems used currently are very often based on voluntary commitments from the player. Therefore, the context in which the gamblers were investigated in the present study had high external (and ecological) validity. However, the reliability is limited due to the fact that data were only collected from one online gambling environment. Replicating the results with other operators and other gambling channels (such as EGMs) would help further corroborate the findings reported here.

It could also the case that players who voluntarily signed up to receive messages about their play were fundamentally different from controls and had a desire and intention to reduce their gambling. Therefore, the control group may not have actually acted as a true control and the impact of messages may have been indirectly inferred rather than measured. Put more simply, gamblers who voluntarily signed up to receive messages may have already been interested in reducing their gambling and would be likely to gamble less. Future studies should also incorporate qualitative information in order to be able to analyses players’ attitudes and opinions toward such systems.

Given this limitation, the authors cannot be certain that it was the intervention that caused the difference in behavior compared to controls, rather than differences in the gamblers who signed up and their motivation to gamble more or less. Simply looking at reductions in time and money spent gambling does not allow the causal mechanism to be determined in the present study. This could only have been done if the players were randomly allocated to receive the informative messages (which was impossible to do given the data were collected on a real gambling site). The present authors have no way of determining if the gamblers read the messages they received and how they were influenced if they were read, or whether it was the personalized feedback and/or the comparative feedback that had most influence in reducing the time and money spent gambling.

The effects of the *mentor* system are fairly modest – 12% above expected for TL and 10% for play duration. Taking this into account, along with the relatively small effect sizes from using the system, some may argue how effective the tool is for reducing the amount of time and money spent gambling. The present authors, while erring on the side of caution, take the more optimistic view that the results do at least suggest that those gamblers using the tool lowered their gambling involvement compared to those not using it. However, other important limitations remain. The results here may not be generalizable to other jurisdictions. Furthermore, the study provides no definitive indication that any of the gamblers who voluntarily opted to use the system were at-risk or problem gamblers. Therefore it is not known whether the system captures gamblers most in need of this intervention. Based on the findings, one explanation may be that the tool may simply be curtailing gambling in those who already gamble responsibly.

A further limitation is that there is no way of knowing whether the target group used other online gambling sites, or land-based gambling, during the evaluation period. These gamblers may have transferred their gambling activity elsewhere to avoid negative personalized feedback via the system (although the present authors think that this is unlikely). Studies such as the British Gambling Prevalence Surveys (Wardle et al., 2007, 2011b) have shown that at-risk and problem gamblers in particular engage with numerous gambling websites and gambling forms. In an ideal study, what is really needed is a pre- and post-assessment of all of these individuals’ gambling, not just the single site. However, this was not possible given the nature of the study.

The fact that this study was performed in a real world setting with objective behavioral data provides many advantages but is limited because motivations and cognitive mechanisms of the participants are unknown and can only be inferred. Furthermore, the study focused on only 2 weeks of gambling behavior following first exposure to the information. Future studies should also examine longer-term behavioral changes and corroborate findings with other psychological ad dispositional mechanisms via the collection of self-report data.

Online gambling operators have the technical capabilities to introduce behavioral feedback systems such as the one described in the present study, and the results presented here suggest that the desired effect of helping players limit the amount of time and money spent gambling can be achieved. Future research should investigate behavioral feedback in more detail in order to better determine which player attributes (e.g., personality traits, beliefs about the nature of games, motivations to gamble, etc.) are associated with positive behavioral changes and whether there are interactions with other variables such as types of games played or intensity of gambling. Furthermore research should focus on investigating personalized messages and at which point in time players should receive messages to optimize behavioral change.

## Conflict of Interest Statement

The authors received no specific funding support for this work. However, the second author has received funding for a number of research projects in the area of gambling education for young people, social responsibility in gambling, and gambling treatment from the Responsibility in Gambling Trust, a charitable body which funds its research programme based on donations from the gambling industry. Both authors undertake consultancy for various gaming companies in the area of social responsibility.

## Footnotes

1 http://www.responsiblegambling.org/en/help/myths.cfm

2 http://www.youtube.com/watch?v=RRIqfAitaoQ

3 http://www.problemgambling.ca/EN/Documents/WinningWaysToKeepGamblingSafe.pdf
